# Systemic Administration of Recombinant Irisin Accelerates Fracture Healing in Mice

**DOI:** 10.3390/ijms221910863

**Published:** 2021-10-08

**Authors:** Silvia Concetta Colucci, Cinzia Buccoliero, Lorenzo Sanesi, Mariella Errede, Graziana Colaianni, Tiziana Annese, Mohd Parvez Khan, Roberta Zerlotin, Manuela Dicarlo, Ernestina Schipani, Kenneth M. Kozloff, Maria Grano

**Affiliations:** 1 Department of Basic Medical Sciences, Neuroscience and Sense Organs, University of Bari, 70124 Bari, Italy; silviaconcetta.colucci@uniba.it (S.C.C.); lorenzo.sanesi@uniba.it (L.S.); mariella.errede@uniba.it (M.E.); tiziana.annese@uniba.it (T.A.); manuela.dicarlo@uniba.it (M.D.); 2 Department of Emergency and Organ Transplantation, University of Bari, 70124 Bari, Italy; cinzia.buccoliero@uniba.it (C.B.); graziana.colaianni@uniba.it (G.C.); r.zerlotin@gmail.com (R.Z.); 3 Departments of Orthopaedic Surgery, University of Pennsylvania, Philadelphia, PA 19104, USA; mohdparvez92@gmail.com (M.P.K.); ernestina.schipani@pennmedicine.upenn.edu (E.S.); 4 Department of Orthopaedic Surgery, University of Michigan, Ann Arbor, MI 48109, USA; kenkoz@umich.edu

**Keywords:** fracture, bone, muscle, chondrocytes, irisin

## Abstract

To date, pharmacological strategies designed to accelerate bone fracture healing are lacking. We subjected 8-week-old C57BL/6 male mice to closed, transverse, mid-diaphyseal tibial fractures and treated them with intraperitoneal injection of a vehicle or r-irisin (100 µg/kg/weekly) immediately following fracture for 10 days or 28 days. Histological analysis of the cartilaginous callus at 10 days showed a threefold increase in Collagen Type X (*p* = 0.0012) and a reduced content of proteoglycans (40%; *p* = 0.0018). Osteoclast count within the callus showed a 2.4-fold increase compared with untreated mice (*p* = 0.026), indicating a more advanced stage of endochondral ossification of the callus during the early stage of fracture repair. Further evidence that irisin induced the transition of cartilage callus into bony callus was provided by a twofold reduction in the expression of SOX9 (*p* = 0.0058) and a 2.2-fold increase in RUNX2 (*p* = 0.0137). Twenty-eight days post-fracture, microCT analyses showed that total callus volume and bone volume were increased by 68% (*p* = 0.0003) and 67% (*p* = 0.0093), respectively, and bone mineral content was 74% higher (*p* = 0.0012) in irisin-treated mice than in controls. Our findings suggest that irisin promotes bone formation in the bony callus and accelerates the fracture repair process, suggesting a possible use as a novel pharmacologic modulator of fracture healing.

## 1. Introduction

Bone fractures have a high incidence in the world population and are often associated with significant disability, imposing high social and health care costs [1]. In 2010, it was estimated that the number of individuals aged >50 years at high risk for osteoporotic fracture worldwide was 158 million, which is expected to double by 2040. The economic burden of fractures has been estimated at 37 billion, with the costs expected to increase by 25% by 2025 [2].

In many cases, fractures heal devoid of adverse outcomes. However, delayed healing may occur in some patients, particularly in those suffering metabolic or vascular disorders. In such cases, surgery is necessary to increase stability and/or promote healing [3,4]. The development of pharmacological agents could provide alternative or additional new approaches to accelerate fracture healing [5].

Fracture healing is a multiphasic process that generally requires months to be completed [6]. Immediately after the initial inflammatory and hematoma phases, the recruitment of mesenchymal progenitor cells leads to the formation of a fibrocartilaginous or soft callus, which is crucial for early stability of the fracture site [7]. As healing progresses, formation of the cartilage callus occurs, and chondroblasts, typically expressing collagen Type II (COL II) matrix protein and transcription factor SRY (sex determining region Y)-box 9 (SOX9), gradually undergo differentiation, lose the expression of the above markers and acquire those typical of hypertrophic chondrocytes, such as collagen Type X (COL X) [8].

The cartilaginous callus turns into the bony callus due to the replacement of cartilage by bone through a process reminiscent of the events occurring during endochondral bone development including the crucial bone-forming activity of osteoblasts, which express the runt related transcription factor 2 (RUNX2). An essential role is also played by osteoclasts, which resorb the cartilage callus and cooperate with osteoblasts for the proper remodeling of the bony callus [9].

Numerous studies, mostly in animal models, have investigated the effect of some bone anabolic agents, such as BMPs, FGFs, activators of the Wnt/βcatenin pathway and PTH/PTHrP receptor agonists [10], in accelerating fracture repair.

Although left unexplored, previous observations have suggested that fracture healing is more efficient when there are muscle flaps present in the area of injury [11]. This pioneering observation was recently brought to light by recent evidence showing that skeletal muscle is an endocrine organ that produces hormone-like molecules, called myokines, with an endocrine/paracrine action on bone tissue [12]. The hormone-like myokine irisin is a newly discovered molecule affecting bone metabolism and it has never been studied as a treatment for fracture healing before.

Irisin, produced by skeletal muscle during exercise, was initially described as crucial for inducing the browning of white adipose tissue, but shortly thereafter, pleiotropic effects emerged in several tissues and organs (bone, muscle, cartilage, pancreas, liver and brain) [13,14]. Our previous studies were the first to prove that the musculoskeletal system is likely to be an important target, as an irisin dose 35 times lower than that effective on adipose tissue, increased bone mass in healthy mice [15], and prevented bone and muscle losses in an osteo-sarcopenic mouse model of disuse-induced osteoporosis and muscle atrophy [16].

These studies showed that irisin treatment in vitro enhanced osteoblast differentiation [17] and improved bone mass in young adult mice in vivo by raising the tibial diaphyseal cross-sectional area due to an enhanced osteoblast activity and bone formation [15]. It was also shown that 100 ng/mL of recombinant irisin directly acts on osteoblasts [17] and osteocytes [18] by activating Erk1/2 phosphorylation and increasing the expression of the Activating Transcription Factor 4 (Atf4), a key transcription factor for osteoblast proliferation, differentiation and survival [15]. In contrast with these results, Kim and colleagues showed that mice with global deletion of the irisin precursor, the Fibronectin Type III Domain Containing 5 (*FNDC5*), were resistant to ovariectomy-induced bone loss through inhibition of osteoclastic bone resorption and osteocytic osteolysis [19]. In vitro data showed that continuous exogenous treatment with 10 ng/mL irisin stimulated osteoclast differentiation from bone marrow precursors. However, increasing the dose to 20 ng/mL had a lower effect on osteoclast number, and doses of irisin equal or exceeding 100 ng/mL decreased osteoclastogenesis [20]. Therefore, it has been hypothesized that the opposing effects of irisin could be due to its concentration, as well as the duration or frequency of treatment. Since its discovery, some reports have questioned the existence of circulating irisin in humans, both because human *FNDC5* has a non-canonical ATA translation start and because of the previous unreliable assay methods used for its detection [21]. In 2015, the study by Jedrychowski and colleagues demonstrated by tandem mass spectrometry that irisin is expressed in humans and is regulated by exercise through the detection and quantification of circulating human irisin at a concentration of ~4 ng/mL [22]. Conversely, another study also using mass spectrometry demonstrated that irisin was not present in human plasma but was detectable in cerebrospinal fluid samples in the range of 0.3 to 1.9 ng/mL [23]. Although quantification of irisin by mass spectrometry should be the gold standard for determining its concentration, this technique requires multi-step sample preparation, such as removal of albumin and immunoglobulins, which could lead to variable amounts of protein being retained for analysis and result in uncontrollable variations in the measurement of irisin [24]. However, since the discovery of irisin, numerous studies have proposed a role of irisin in multi-organ protection in humans, and much progress has also been made to demonstrate its key role as one of the determinants of skeletal status. Human studies have shown that postmenopausal women with previous osteoporotic fractures had low irisin levels [25] and that circulating irisin was inversely related to vertebral fragility fractures [26]. Furthermore, previous work demonstrated a positive association between circulating irisin levels and bone mineral status in adult and child populations [27,28,29,30]. More recently, it was reported that low concentrations of irisin in older women were associated with an increased risk of hip fractures [31].

Notably, the levels of the irisin precursor, *FNDC5*, in the skeletal muscle of the older adult subjects were positively associated with irisin serum levels and *osteocalcin* mRNA expression in bone biopsies, indicating a strong correlation between healthy muscle and bone tissues [29].

Moreover, recent findings highlighted that irisin also targets cartilage. An in vitro study of human osteoarthritic chondrocytes showed that the myokine directly affects chondrocytes and improves cellular anabolism while decreasing their catabolism [32]. Additionally, irisin signaling was required to protect against oxidative damage, apoptosis and extracellular matrix underproduction in inflamed chondrocytes, delaying osteoarthritis development [33].

In light of the action of irisin as a powerful stimulant of bone and cartilage growth, we hypothesized that an irisin-based treatment could improve the fracture healing of long bones in mice.

In testing our hypothesis, this study is the first to identify the positive effect of irisin on fracture healing by accelerating the shift from cartilage callus to bony callus in a mouse model of tibial fracture.

## 2. Results

### 2.1. Irisin Induces Maturation of the Soft Callus at 10 Days Post-Fracture

**Figure 1:**
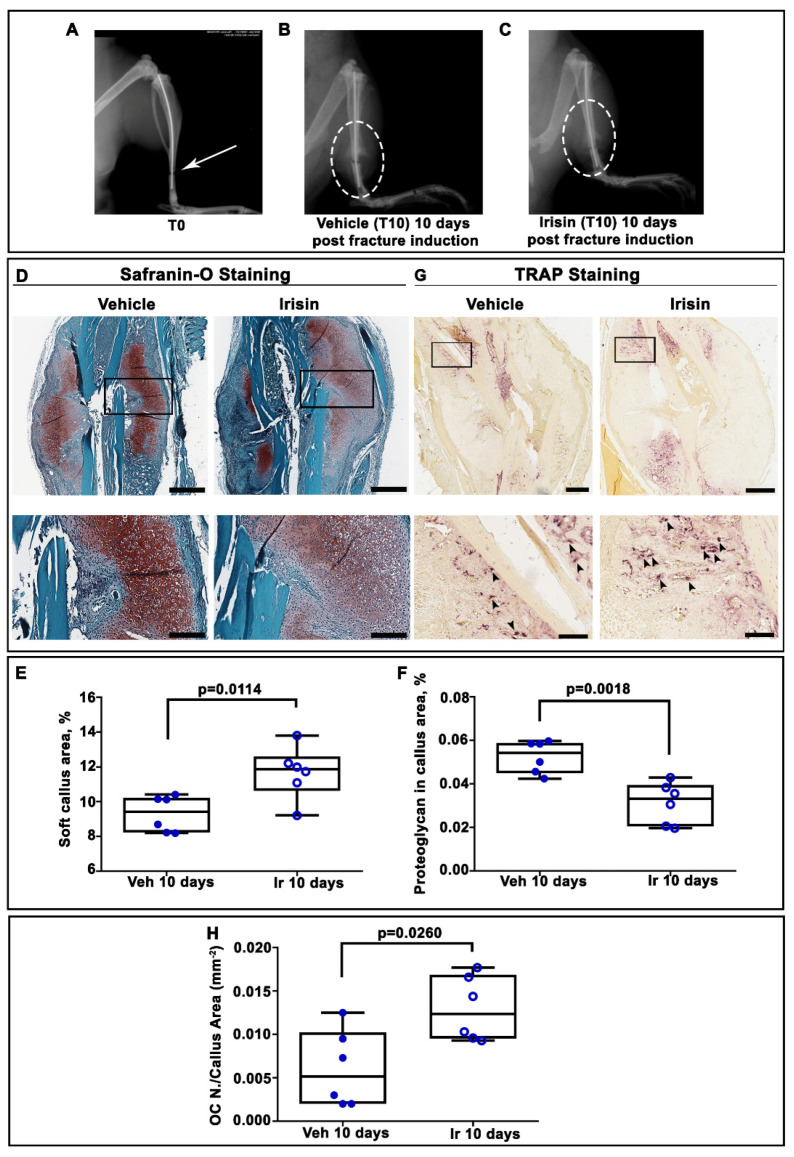
(**A**) Representative radiological images of fractured tibia at Time 0 (T0), and mice intermittently treated with (**B**) normal saline (vehicle) or (**C**) irisin at 10 days post-fracture. (**D**) Representative Safranin O-staining images of callus sections from vehicle- and irisin-treated mice at 10 days post-fracture (scale bar 0.8 mm). The black squares indicate the areas of higher magnification (scale bar: 60 μm). (**E**) Dot-plot graphs showing the increased soft callus area and (**F**) decreased proteoglycan-rich cartilage matrix in irisin-treated mice (*n* = 6) compared with vehicle-treated mice (*n* = 6). (**G**) Representative Trap staining images of callus sections from vehicle- (*n* = 6) and irisin-treated (*n* = 6) mice at 10 days post-fracture (scale bar: 0.8 mm). The black squares indicate the areas of higher magnification. (**H**) Dot-plot graph showing the significantly increased number of Trap-positive cells in the callus area (OC *n*. /CA) in irisin-treated mice (*n* = 6) compared with vehicle-treated mice (*n* = 6) (scale bar: 60 μm). Data are presented as dot-plots with medians, from maximum to minimum, with all data points shown. The Mann–Whitney test was used to compare groups.

X-ray radiography performed directly after surgery confirmed transverse mid-diaphyseal tibial fractures and the adequate positioning of intramedullary pins (Figure 1A). Serial radiographs of representative mice, intermittently treated with normal saline (vehicle) or irisin, showed clearly visible fracture lines in both treatment groups at 10 days post-fracture (Figure 1B,C). To determine the total cartilage area within the soft callus, Safranin-O staining was performed at the same time point (Figure 1D). Histological analysis revealed increased callus area but lower proteoglycan content in the soft callus of irisin-treated mice (Figure 1D). The histological observation was confirmed by morphometric analysis of the entire callus, showing a significantly higher percentage of soft callus area (+25%; *p* = 0.0114) but lower proteoglycan content (−40%; *p* = 0.0018) in irisin-treated mice compared with control mice. Furthermore, tartrate-resistant acid phosphatase (Trap)-positive osteoclasts within the callus tissue were also assessed by histology (Figure 1G). Quantification of Trap-positive (Trap+) cells within the callus area showed a 2.4-fold increase in osteoclast numbers on the callus area (OC N. /CA) at 10 days (*p* = 0.026) after fracture in irisin-treated mice (Figure 1H), thus suggesting a different stage of soft callus formation following treatment with irisin. To decipher the factors involved in irisin-induced cartilaginous callus formation, we performed an immunohistochemical analysis of the matrix proteins and transcription factors expressed by the chondrocytes during their progression to the hypertrophic phenotype.

**Figure 2:**
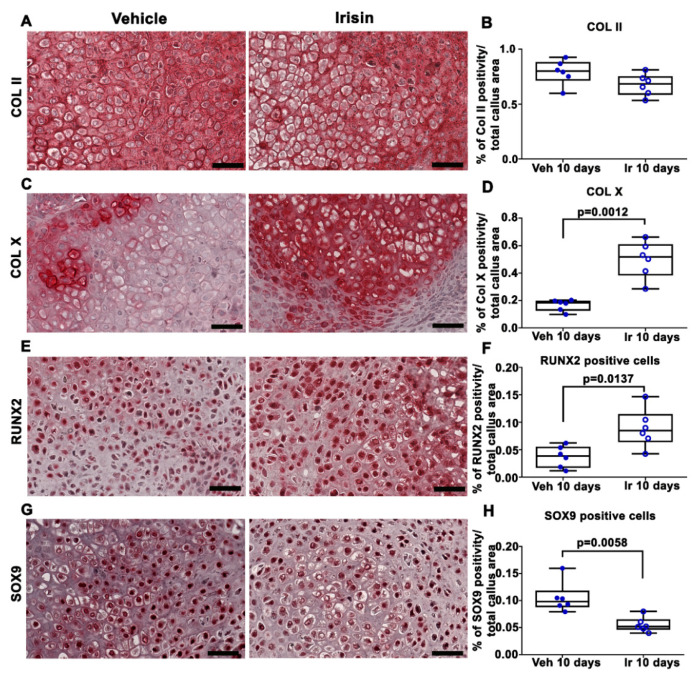
Representative images of (**A**) COL II, (**C**) Col X, (**E**) RUNX2 and (**G**) SOX9 immunostaining in callus sections from vehicle-treated mice (*n* = 6) and irisin-treated mice (*n* = 6) at 10 days post-fracture (scale bars: 20 µm). Dot-plot graphs showing the quantification of (**B**) COL II, (**D**) COL X, (**F**) RUNX2 and (**H**) SOX9 expression. Data are presented as dot-plots with medians, from maximum to minimum, with all data points shown. The Mann–Whitney test was used to compare groups.

Immunohistochemical staining for COL II in callus sections (Figure 2A) and relative quantification (Figure 2B) showed no significant difference between the two experimental groups. However, the expression of COL X, a well-established marker of hypertrophic chondrocytes, was increased threefold (*p* = 0.0012) in the callus of irisin-treated mice compared with the vehicle group (Figure 2C,D). Of note, the positivity for the master regulator of osteoblast differentiation, RUNX2, was 2.2-fold higher (*p* = 0.0137) (Figure 2E,F), whereas the positivity for SOX9, the transcription factor that regulates chondrogenesis, was twofold lower (*p* = 0.0058) (Figure 2G,H) in the callus of irisin-treated mice than in the vehicle-treated group.

### 2.2. Irisin Increased Bony Callus Size at 28 Days Post-Fracture

**Figure 3:**
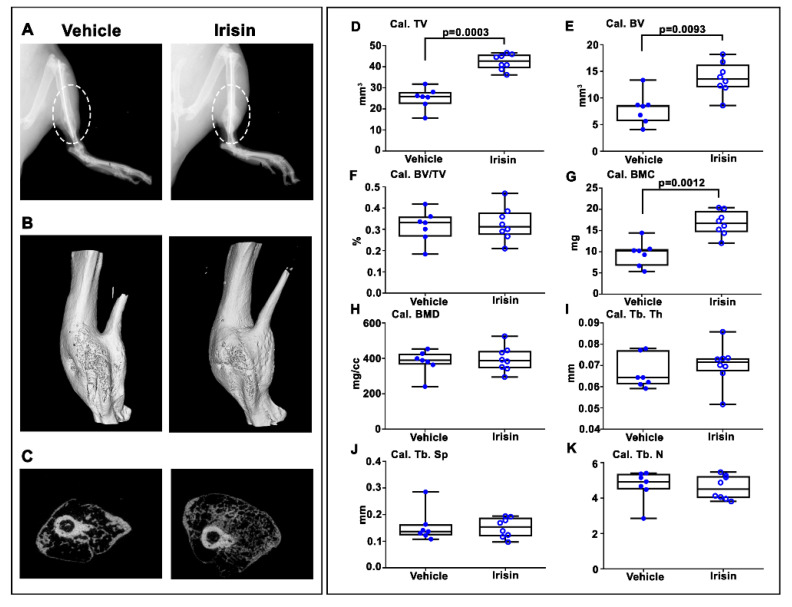
Mice sacrificed at 28 days post-fracture. (**A**) Representative radiological images. (**B**) Longitudinal and (**C**) cross-sectional micro-computed tomography (μCT) 3D reconstructions of fractured tibiae from vehicle- and irisin-treated mice at 28 days post-fracture. µCT analysis of the bone callus: (**D**) Cal. TV= callus total volume; (**E**) Cal. BV= callus bone volume; (**F**) Cal. BV/TV= callus bone volume/total volume; (**G**) Cal. BMC = callus bone mineral content; (**H**) Cal. BMD = callus bone mineral density; (**I**) Cal. Tb. Th = callus trabecular thickness; (**J**) Cal. Tb. Sp = callus trabecular separation; (**K**) Cal. Tb. *n*= callus trabecular number. Vehicle-treated mice (*n* = 7); irisin-treated mice (*n* = 8). Data are presented as box-and-whisker plots with median and interquartile ranges, from maximum to minimum, with all data points shown. The Mann–Whitney test was used to compare groups.

After 28 days post-fracture, X-ray images showed that callus was still evident in both vehicle- and irisin-treated mice (Figure 3A). However, longitudinal and cross-sectional micro-computed tomography (microCT) 3D reconstructions (Figure 3B,C) clearly indicated an increased callus size in the tibia of irisin-treated mice. Due to the absence of mineralization of the callus at 10 days post-fracture, microCT analysis was performed only on the callus at 28 days post-fracture. Callus total volume (Cal TV) (Figure 3D) and callus bone volume (Cal BV) (Figure 3E) increased by 68% (*p* = 0.0003) and 67% (*p* = 0.00193), respectively, in irisin-treated mice compared with the control group, resulting in an unchanged callus bone volume fraction (Cal. BV/TV) (Figure 3F). Moreover, the bone mineral content of the callus (Cal. BMC) (Figure 3G) was 74% higher (*p* = 0.0012) in irisin-treated mice than in the controls, whereas the callus bone mineral density (cal. BMD) (Figure 3H) was unchanged. Consistent with the unchanged bone volume fraction in the callus, measurements of trabecular parameters showed that there was no significant difference in trabecular thickness (Cal. Tb. Th) (Figure 3I), trabecular separation (Cal. Tb. Sp) (Figure 3J) and trabecular number (Cal. Tb. N) (Figure 3K) between the two groups of mice.

### 2.3. Irisin Accelerated Bony Callus Formation at 28 Days Post-Fracture

**Figure 4:**
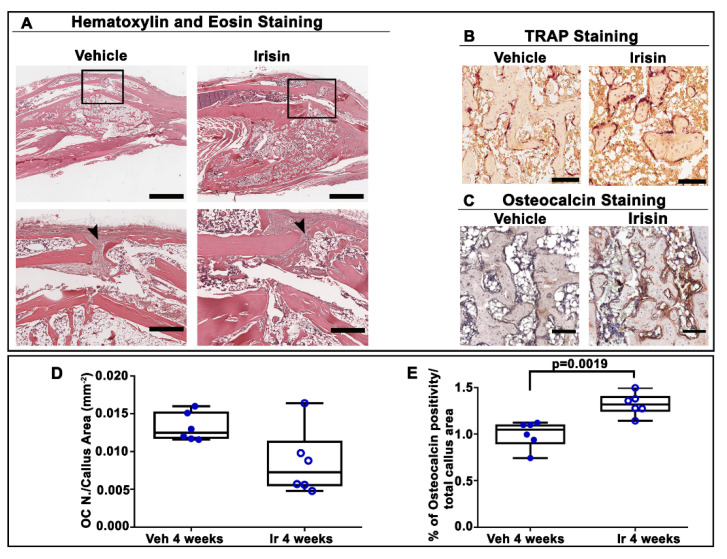
(**A**) Representative hematoxylin and eosin staining images on callus sections from vehicle- and irisin-treated mice at 28 days post-fracture (scale bar: 0.8 mm). The black squares indicate the area of higher magnification (scale bar: 60 µm). Arrowheads indicate the fibrous tissue. (**B**) Representative Trap staining images of callus sections from vehicle- and irisin-treated mice at 28 days post-fracture (scale bar: 30 µm). (**C**) Representative images of osteocalcin immunostaining in callus sections from vehicle- and irisin-treated mice at 28 days post-fracture (scale bar: 30 µm). (**D**) Quantification of OC N./CA in sections from vehicle- (*n* = 6) and irisin-treated mice (*n* = 6) at 28 days post-fracture. (**E**) Quantification of the percentage of osteocalcin-positive cells (osteocalcin^+^ cell, %) out of all cells of the callus from vehicle-treated mice (*n* = 6) and irisin-treated mice (*n* = 6) at 28 days post-fracture. Data are presented as dot-plots with medians, from maximum to minimum, with all data points shown. The Mann–Whitney test was used to compare groups.

To further characterize the influence of irisin treatment on the structural organization of the bony callus at 28 days post-fracture, we performed histological and immunohistochemical analysis of callus sections. Hematoxylin and eosin staining showed that irisin promoted the formation of bony callus (Figure 4A), thereby accelerating fracture healing. In contrast, in vehicle-injected mice, the fibrous tissue was still prevalent in the fracture gap. Trap staining (Figure 4B) of callus sections and the relative morphometric analysis (Figure 4D) showed the decreasing, although not significant, trend of the osteoclast number in the callus area (OC N./CA) in irisin-treated mice compared with the vehicle group. Interestingly, immunohistochemistry for osteocalcin (Figure 4C) revealed a higher percentage of positive cells for this bone matrix protein (+26%; *p* = 0.0019) (Figure 4E) in irisin-treated mice, thus suggesting that irisin promotes bone formation in the bony callus, possibly improving the fracture repair process.

## 3. Discussion

The process of fracture healing, from the fracture event to its repair, recapitulates events that occur during skeletal development, particularly in the process of endochondral ossification [6]. It is well established that this dynamic process is triggered immediately after a fracture by the secretion of cytokines capable of recruiting mesenchymal stem cells that differentiate into chondrocytes to form the fibrocartilaginous callus and ultimately into osteoblasts to build new bone tissue [34]. Our data shown here clearly demonstrate that pharmacologic treatment with the muscle-derived irisin of mice with a fracture accelerated the transition from cartilaginous to bony callus and stimulated the deposition of new mineralized matrix.

The efficacy of irisin in increasing the rate of healing was already evident during the cartilage phase of repair, at Day 10 post-fracture. At this time point of the repair process, we observed an increase in collagen Type X expression, which indicated that the transition of the chondrocytes to their hypertrophic phenotype was accelerated by irisin treatment. The transition from cartilaginous to bony callus was further confirmed by the reduction in SOX9 expression, a key transcription factor of chondrocytes [35], and by a concomitant increase in RUNX2, the most important transcription factor regulating osteoblast differentiation [36,37].

Most notably, histological analysis of the tibiae at Day 10 post-fracture showed an irisin-induced increase in the soft callus area associated with a reduction in proteoglycan content.

These results are in line with the modification of the gene expression pattern of chondrocytes towards the hypertrophic phenotype, which allows them to modify the composition of the cartilage matrix [38]. Accordingly, we observed a trend towards a decrease in collagen Type II, associated with a marked increase in collagen Type X. Therefore, the decrease in proteoglycan content might depend on a more rapid irisin-mediated degradation of the cartilage matrix, as hypertrophic chondrocytes activate the selective secretion of matrix metalloproteinase 13, a collagenase active in degrading collagen Type II fibrils [39,40].

Additionally, we found a higher percentage of osteoclasts within the callus area in irisin-treated mice, thus implying acceleration towards the callus remodeling phase.

Observable differences in callus size were also detected at Day 28 post-fracture, presumably soon after new endochondral bone formation. There were overall increases in callus volume (+67%) and total callus mineral content (+74%), but not a higher average mineral density. It is possible that the parallel increase in callus volume and mineral content produced a more stable fracture site. Furthermore, consistent with unchanged BV/TV in the callus, measurements of trabecular parameters such as trabecular number, thickness and separation between the two groups of mice demonstrated that subsequent bone remodeling was relatively normal after irisin treatment. Our observations are in line with previous data showing that systemic injections of the anabolic parathyroid hormone PTH(1–34) in mice with fractures induced a larger callus cross-sectional area, and increased the length (+30%), total volume (+83%) and bone mineral content (+60%) without affecting the trabecular microarchitecture within the callus. Moreover, similar to our study, chondrocyte hypertrophy in the callus occurred earlier in PTH-treated mice [41]. There are numerous similarities between irisin and PTH, and we previously showed that, under physiological conditions, these two hormones mutually control each other through negative feedback [42]. Treatment with 1–34 PTH (teriparatide) in myotubes in vitro downregulated the expression of the irisin precursor, *FNDC5* [42]. In parallel, irisin treatment decreased the expression of the PTH receptor in osteoblasts in vitro [42], suggesting that irisin might exert its anabolic function on bone not only by stimulating osteoblast activity but also by reducing the action of PTH on these cells. Both irisin and PTH decrease the expression of sclerostin, the most potent inhibitor of the anabolic Wnt pathway [16,43], and prevent osteocyte apoptosis by modulating the expression of Atf4 in these cells [18,44]. Finally, as for PTH, which exerts both catabolic and anabolic effects on the skeleton, depending on the administration regimen [43], high doses of irisin can lead to bone catabolism [19], whereas lower doses, given in intermittent pulses, as occurs during exercise, can have anabolic effects on bone [15]. More specifically, Kim et al. [19] observed increased expression of sclerostin, an inhibitor of bone formation, after six daily injections of 1 mg/kg of irisin. In contrast, a reduction in sclerostin was observed by injecting mice with a dose 10 times lower (100 μg/kg), given weekly for 4 weeks [15]. Moreover, Estell and colleagues demonstrated that irisin administered at 10 ng/mL stimulated osteoclast formation in vitro and in bone marrow progenitors of *FNDC5* transgenic mice [20]. Although apparently in contrast to our results, it is important to note that type of recombinant irisin, the dose and the duration of its treatment are crucial factors in the cellular response, and these aspects may be responsible for the discrepancies observed in the different studies [20].

In order to further understand the structural organization of the callus after 28 days of intermittent irisin treatment, we performed histological and immunohistochemical analyses. The increase in bone callus volume, as observed by microCT analysis, was confirmed by hematoxylin and eosin staining, demonstrating that irisin promoted bone fracture healing at a faster rate than vehicle treatment, as previously demonstrated in PTH-treated mice [41]. Notably, a more abundant bone matrix at the fracture site following irisin treatment was confirmed by the increased expression of osteocalcin, the bone matrix protein involved in activation of the mineralization process [45]. Hence, the increased production of osteocalcin would explain the increase in mineral content as detected by microCT analysis. Abundant evidence has shown that osteocalcin plays a key role in the bone matrix by bridging hydroxyapatite crystals with osteopontin, which, in turn, binds collagen fibers [46], and provides adhesive support for osteoblasts and osteoclasts [47]. It has been hypothesized that, following bone injury, the presence of osteocalcin on the organic–inorganic interface of the bone matrix allows more energy to be dissipated on collagen fibers, providing an important contribution to bone fracture resistance [48]. Furthermore, we previously highlighted the existence of a link between irisin and osteocalcin. In a population of older adult subjects, we observed that *osteocalcin* expression in bone biopsies was positively associated with the irisin precursor, *FNDC5*, expressed in skeletal muscle biopsies [29]. In agreement with these findings, in vitro data showed that treatment with recombinant irisin increased osteocalcin expression in primary mouse and rat osteoblasts, and in dental bud stem cells undergoing osteogenic differentiation [15,49,50].

Identifying novel therapeutic strategies that stimulate bone regeneration has the potential to significantly improve outcomes in fracture healing, especially in conditions in which several risk factors coexist that may alter this process, including aging, osteoporosis and comorbidities characterized by impaired bone metabolism that negatively affect fracture repair. Currently, the use of novel pharmacological agents is being explored for fracture healing [10], as the bone morphogenetic protein-2 is the only US FDA-approved therapeutic application to be used post-fracture. Unfortunately, due to its high cost and the very narrow window of administration (within 14 days of injury), the use of this medication is intended only for the most serious fractures [51].

Overall, our results show that systemic administration of intermittent low doses of irisin accelerates bone fracture healing in mice by promoting bone formation and mineralization. The transcription factor and matrix component expression analysis, histomorphometry and microCT data together demonstrate that irisin during fracture repair stimulates osteogenesis to produce more bone tissue that can stabilize the fracture more rapidly without altering the normal process of bone remodeling. Nevertheless, this study lays the basis for the use of recombinant irisin in fracture repair by providing a complementary analysis of tibial callus tissue following systemic irisin administration. Most importantly, we have added new data that increase our understanding of the processes that regulate and promote the conversion of cartilage to bone during fracture repair. Speculatively, our results may also provide a possible explanation for why bone fractures heal faster when muscle flaps are present at the fracture site: it could be irisin, produced by the muscle cells, that mediates this effect.

Finally, one of the key roles of irisin as an anti-inflammatory molecule should also be considered. The study by Narayanan and colleagues showed that exogenous treatment with irisin, given i.p. at 18 ng/mL twice a week for 3 weeks, reduced the expression of tumor necrosis factor-alpha (TNF-α) in rats with inflammatory bowel disease [52]. It is known that TNF-α is recognized as a primary mediator in the inflammatory reaction that initiates the reparative process of fractures. Immediately after fracture, TNF-α is elevated and then decreases to normal physiological levels for most of the reparative process [53]. However, if TNF-α levels remain elevated, this will affect fracture healing negatively, leading to higher soft tissue in the callus and decreased biomechanical bone stability [54]. Therefore, it is highly relevant that future studies should seek to understand the influence of irisin treatment on changes in TNF-α levels during fracture repair.

This will open new avenues for the exploration of the mechanisms by which irisin acts on bone and cartilage tissue and the identification of molecules that improve fracture healing. Despite these promising results gained in the murine model of fracture, the transfer of such findings to humans will require careful additional evaluation. The fracture healing process is very similar in mice and humans, making the mouse a good model for fracture [55]. However, the effect of irisin in the fracture healing process in humans will need further study, since differences between humans and mice have been observed in some physiological processes, such as the browning response in adipose tissue [56].

There is an urgent need for the generation of new therapies that could especially benefit delayed fracture repair or non-union cases, which occur in 2–5% of the normal population but can reach 50% in fracture cases in the population of individuals with metabolic or vascular disorders. The new evidence provided here encourages further investigation to confirm the promising potential of irisin as a new therapy for fracture repair.

## 4. Materials and Methods

### 4.1. Experimental Design and Tibial Fracture Procedure

Experiments were performed on 8-week-old C57BL/6 male mice. Mice were housed in standard rodent cages and maintained under standard conditions on a 12/12-h light/dark cycle with access to water and a regular chow diet ad libitum (Harlan Teklad 2019, SDS, England).

**Figure 5:**
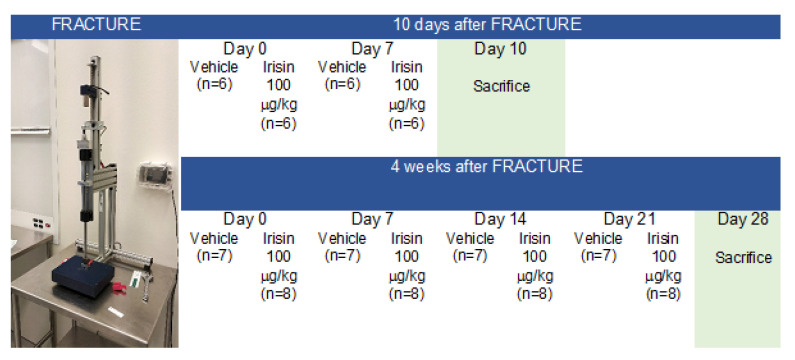
Experimental plan.

Closed transverse mid-diaphyseal tibial fractures were created on the right tibia in each mouse, similar to previously published methods [57,58]. Mice were anesthetized with isofluorane gas and a small incision was made medially to the tibial tuberosity. The bone cortex of the tibial plateau was punctured using a 26-gauge needle, and a 0.22 mm sterile diameter pin was inserted through the length of the intramedullary canal. Fractures were created using a custom guillotine device (Figure 5). The incision was closed with surgical sutures. Buprenorphine (0.05 mg/kg) was administered subcutaneously pre- and post-operation. Carprofen (5 mg/kg) was administered immediately post-operation and during the recovery period. Post-surgery X-ray scans were generated using a microradiography system (Faxitron, Wheeling, IL, USA) to verify the fracture position and proper pin placement.

Of the 32 mice undergoing fracture induction, *n* = 5 mice with a displaced fracture were excluded from post-fracture treatment. Mice were randomly divided into two groups: one group (*n* = 6, vehicle treatment; *n* = 6, irisin treatment) was sacrificed 10 days after fracture induction, and the other group (*n* = 7, vehicle treatment; *n* = 8, irisin treatment) was sacrificed 28 days after fracture induction, as described in the experimental plan (Figure 5).

As shown in Figure 5, immediately following the fracture, mice were treated weekly for 10 days or 28 days via intra-peritoneal (i.p.) injection with a vehicle or 100 µg/kg of untagged recombinant irisin produced in *E. coli* (Adipogen International, San Diego, CA, USA) and previously validated by ELISA, which demonstrated that it was preserved in the cell culture medium for up to 48 h when administered to MLO-Y4 cells [18].

After the pre-established healing periods, euthanasia was performed and bone segments were fixed 72 h in PFA 4%. All animal experiments described in this article were reviewed and approved by the University of Michigan’s Committee on Use and Care of Animals Protocol #PRO00008779 (Goldstein).

### 4.2. X-ray and Micro-Computed Tomography

X-ray scans were collected using a Faxitron X-Ray. X-ray scans were taken immediately after euthanasia to observe callus conformation at 10 days (vehicle-treated mice, *n* = 6; irisin-treated mice, *n* = 6) and 28 days (vehicle-treated mice, *n* = 7; irisin-treated mice, *n* = 8).

All fracture calluses and contralateral non-fractured tibiae were dissected free from attached muscle and the intramedullary pins were removed. All samples were stored in 70% ethanol. The tibiae at 28 days post-fracture (vehicle-treated mice, *n* = 7; irisin-treated mice, *n* = 8) were scanned using an eXplore Locus SP microCT system (GE Healthcare, London, ON, Canada). Scanning parameters included a 80 kVp and 80 μA X-ray source, a rotation angle with 0.5° increments and a 1600-millisecond exposure. To reduce beam-hardening artifacts, the tibiae were immersed in distilled water, and a 0.02-inch aluminum filter was used with an acrylic beam flattener around the tibiae. Images were reconstructed to an isotropic voxel size of 18 μm and calibrated using a hydroxyapatite phantom. Images were analyzed and quantified using Microview Software (Parallax Innovations, Ilderton, ON, Canada). The callus area was analyzed without existing cortical bone. Due to the absence of mineralization of the callus in the 10-day-old calluses, only 28-day fracture calluses were analyzed by microCT. MicroCT scans were reoriented for analysis and snapshots of the callus were captured. Callus and cortical bone sections were manually identified in the first slice and then had spline interpolation between points. On average, 600 slices were analyzed over a tibia length of approximately 6 mm, corresponding to the callus area. The points were chosen every 5 slices. The cortical bone sections were removed from the image to analyze the callus only. A fixed threshold of 1600 Hounsfield units was applied to calculate the callus and bone parameters following the recommendations of the ASBMR guidelines [59].

MicroCT reconstructions were performed to obtain the following parameters: callus bone volume (Cal.BV), callus bone mineral density (Cal. BMD), callus total volume (Cal. TV), callus BV/TV (Cal.BV/TV), callus bone mineral content (Cal. BMC), callus trabecular thickness (Cal. Tb. Th), callus trabecular number (Cal. Tb. N) and callus trabecular separation (Cal. Tb. Sp).

### 4.3. Histological and Immunohistochemical Assays

At 10 days (*n* = 12) and 28 days (*n* = 12), fractured tibiae were dissected and disarticulated from the knee, with the surrounding muscles removed, then treated for histology and histomorphometric analysis. Fractured tibiae were decalcified with EDTA at 20% and pH 7.5, embedded in paraffin and cut into 5 μm sections on a standard microtome (RM-2155 Leica, Heidelberg, Germany). Sections collected from 10-day fractured tibiae from each mouse (vehicle, *n* = 6; irisin, *n* = 6) were stained with Safranin-O (Merck Millipore, Danvers, MA, USA), an orthochromatic dye that selectively identifies cartilage sulfated glycosaminoglycans, and counterstained with Fast Green FCF (Merck Millipore). Moreover, in 10-day old callus, immunohistochemistry was performed using the Dako REALTM Detection System Alkaline Phosphatase/RED Rabbit/Mouse (K5005 Dako, Santa Clara, CA, USA). Sections were incubated with Coll II (MAB8887, Sigma-Aldrich, St. Louis, MO, USA), Coll X (ab260040, Abcam, Cambridge, UK), Runx2 (ab192256 Abcam) and Sox9 (ab185966, Abcam) primary antibodies (vehicle, *n* = 6; irisin, *n* = 6). In addition, both 10-day and 28-day fractured tibiae sections were stained using a tartrate-resistant acid phosphatase (Trap) kit (Sigma-Aldrich, St. Louis, MO, USA) for osteoclast quantification (vehicle, *n* = 6; irisin, *n* = 6). The number of osteoclasts (OC *n*.), as Trap+ multinucleated cells (more than three nuclei), was counted in the total callus area (CA mm^2^), measured as explained in the morphometric analysis paragraph by ImageScope software (Leica Biosystems, Nussloch, Germany). Furthermore, sections of 28-day fractured tibiae were stained with hematoxylin and eosin for observing the bony callus area immunostained with the anti-osteocalcin (sc-365797, Santa Cruz, CA, USA) primary antibody to study its expression in all cells on callus bone surfaces (vehicle, *n* = 6; irisin, *n* = 6).

### 4.4. Morphometric Analysis

Stained sections were digitalized using the Aperio ScanScope CS (Leica Biosystems) whole-slide scanning platform at the maximum magnification (400×) available and stored as high-resolution digital images on the workstation associated with the instrument. Morphometric analysis was performed by two independent observers on two adjacent selected sections from each side of each callus’s widest area. A total callus area of 13.5 mm^2^, corresponding to the widest callus area (region of interest, ROI) of all callus sections, was analyzed using the Aperio Color Deconvolution algorithm included in the ImageScope v.11.2.0.780 (Leica Biosystems). The algorithm allowed us to distinguish the various colors (callus and different IHC markers) of the stained tissue images, converting them into separate digitalized channels. Each single-color channel was then analyzed by applying the Aperio Positive Pixel Count algorithm, which was set to detect the number of strong positive pixels (Nsp), medium positive pixels (Np) and weak positive pixels (Nwp) [60]. The percentages of total positive pixels were reported in graphs.

### 4.5. Statistical Analysis

All data are presented as boxplots with medians, interquartile ranges, and maximum and minimum values. All variables were checked for normality (Shapiro–Wilk normality test) to see the data distribution. For parameters with a normal distribution, mean values were compared using Student’s *t*-test; otherwise, for parameters that were not normally distributed, significance was evaluated with Mann–Whitney U-test using GraphPad Prism (GraphPad Software, Inc., La Jolla, CA, USA). Differences were considered significant at *p* < 0.05.
